# AGEISM, JOB SATISFACTION, INTRINSIC MOTIVATION, AND RETIREMENT DECISIONS

**DOI:** 10.1093/geroni/igad104.0204

**Published:** 2023-12-21

**Authors:** Hannah Swift

**Affiliations:** University of Kent, Canterbury, England, United Kingdom

## Abstract

This study examines experiences of ageism in the workplace and how they impact workers’ intentions to retire. Self-report data on experiences of ageism in the workplace, meta-perceptions of older workers, job satisfaction, motivation, and retirement intentions were collected in a sample of 125 UK employees aged 50 and older, alongside other relevant demographic information. In line with previous research, the analysis revealed that the positive relationship between ageism and intentions to retire is mediated by a reduction in job satisfaction. However, additional analysis confirmed that intrinsic motivation also partially mediates the relationship between job satisfaction and intentions to retire. A serial mediation model then established that experiences of ageism at work increased intentions to retire via reductions in job satisfaction, which in turn reduced intrinsic motivation to work. Separate models confirm these relationships remain when the experience of ageism is substituted for negative meta-perceptions of older workers. This research provides further evidence for the need to reduce ageism and negative age stereotypes in organizational settings but also highlights the role of intrinsic motivation as an important indirect pathway through which ageism increases retirement intentions. For the first time, the effects of negative meta-stereotypes on job satisfaction, intrinsic motivation, and intentions are also modelled.

